# *In vitro* assessment of cytokine release from gingival fibroblasts exposed to periodontal pathogens

**DOI:** 10.6026/973206300211501

**Published:** 2025-06-30

**Authors:** Kaushal Pati Tripathi, Rohit Mishra, Varsha Choubey, Ajay Kumar, Arohi Aglawe, Anshul Gulati

**Affiliations:** 1 Department of Periodontics & Implantology, Hitkarini Dental College & Hospital, Jabalpur 482005, Madhya Pradesh, India; 2 Department of Oral Medicine & Radiology, Faculty of Dental Sciences, Institute of Medical Sciences, Banaras Hindu University, Varanasi-221005, Uttar Pradesh, India; 3 Department of Periodontology, Mansarovar Dental College, Kolar Bhopal, Madhya Pradesh-462042, India

**Keywords:** Gingival fibroblasts, periopathogens, inflamed gingiva, cytokines

## Abstract

Cytokine responses of gingival fibroblasts from healthy and inflamed tissues upon exposure to *P. gingivalis* *lipopolysaccharide* (LPS) are of interest. Samples were obtained from 20 individuals undergoing periodontal procedures. Inflamed fibroblasts showed elevated secretion of IL-6, MCP-1, IL-8, and GRO. *P. gingivalis* LPS induced a stronger cytokine response than controls. Thus, inflamed fibroblasts can mount a secondary inflammatory response to periodontal pathogens.

## Background:

When Gram-negative microorganisms like *Porphyromonas gingivalis* (*P. gingivalis*) and a variety of other pathogenic organisms, among which are *Treponema denticola* alongside *Fusobacterium nucleatum*, contaminate the subgingival surroundings, the host's immune system response triggers the emergence of periodontitis [1, 2-3]. *P. gingivalis* is an anaerobic bacterium that belongs to the red complex group of bacteria. It is a crucial microorganism linked to periodontitis and its clinical manifestations [4, 5- 6]. The response of the immune system to the initial contact between *P. gingivalis* and various kinds of cells, such as gingival epithelial cells, gingival fibroblasts (GF), tissue-resident macrophages, as well as moving pro-inflammatory neutrophils, monocytes and macrophages and may influence the onset and development of periodontitis [7, 8-9]. Numerous pro-inflammatory mediators, such as tumor necrosis factor (TNF), Interleukin-1 (IL-1), IL-8 and IL-6 are produced by cells in the gingival tissue [10, 11-12]. In an attempt to manage the resulting infection and preserve the equilibrium of the equilibrium of the tissues, these cytokines play a role in attracting and stimulating inflammatory cells, thereby permitting the regeneration of tissue and healing [13, 14-15]. The overall inflammation linked to periodontitis is also influenced by circulating inflammatory cells. Changes in circulation levels of inflammatory mediators caused by sub gingival contamination by pathogenic bacteria in periodontal tissue increase the likelihood of developing several chronic diseases, including rheumatoid arthritis, heart disease, diabetes and reinforce the association between periodontitis and these conditions [16, 17-18]. Chronic, unresolved inflammation damages the tissues that support the periodontal cavity if the immune system's response to periodontal infections is not adequately managed [17, 18- 19]. Once believed to be harmless spectators in the host defense system, gingival fibroblasts (GFs) are now understood to actively and significantly contribute to the coordination of numerous defense systems required to fight infection [9, 10-11]. *lipopolysaccharide* (LPS), one of the several virulence factors of *P. gingivalis*, causes these cells to react metabolically and inflammatorily. It is believed that *P. gingivalis* *lipopolysaccharide* interacts with gingival fibroblasts through Toll-Like Receptors (TLR) and triggers several intracellular signaling pathways, which in turn modulates the production of cytokines [8, 10]. CD14 is also expressed on the cell surface by gingival fibroblasts [13, 14- 15], but it lacks an intracellular signaling moiety that would enable messages to reach the cell [16, 17-18]. Even though several research have shown that gingival fibroblasts react to *P. gingivalis* LPS, most of them use cells obtained from donors that are in good health, readily accessible gingival fibroblasts cell lines, or murine cell lines [13, 14, 15, 16-17]. Few studies have used gingival fibroblasts from inflamed gingiva for inclusive investigation. The generation of inflammatory cytokines by *P. gingivalis* *lipopolysaccharide* is most frequently contrasted with the effect of *E. coli* *lipopolysaccharide* exposure in studies conducted to evaluate gingival fibroblasts cytokine production [12, 13, 14- 15]. Therefore, it is of interest to compare the cytokine response of primary gingival fibroblasts from healthy and inflamed gingival tissues and to evaluate whether gingival fibroblasts from inflamed gingiva are accomplished of establishing a secondary inflammatory response after exposure to *P. gingivalis* *lipopolysaccharide* *In vitro*.

## Methods and Materials:

## Gingival tissue collection and culture:

10 individuals undergoing crown lengthening procedure had samples of healthy gingival tissue taken, and 10 individuals who had a clinical diagnosis of chronic periodontitis had samples of inflamed tissue taken after flap debridement. Gingival tissues were examined histologically in accordance with earlier reports [25]. In summary, half of each tissue sample-five inflamed and five healthy-was fixed for four hours in 4% paraformaldehyde, washed for two hours under running water, dried and then embedded in paraffin. Before cutting 4µm thick serial slices and performing standard staining with hematoxylin and eosin, blocks were kept at 4°C. In order to verify the clinical diagnosis of either healthy or inflamed tissue, the haematoxylin and eosin sections were examined under a light microscope. This was done by looking for signs of lymphocyte and plasma cell infiltration in the gingival connective tissue.

## Gingival fibroblast cell treatments:

A dosage response and time course was performed to ascertain the best way to stimulate cytokines. Healthy gingival fibroblasts were cultivated overnight to achieve 80% confluence after being seeded at a density of 5 x 104 cells/well in 24-well tissue culture plates. At this point, the culture medium was taken out, the monolayer was cleaned with Hanks Balanced Salt Solution, serum-free media was added and the mixture was cultured for twenty-four hours. After that, cells were subjected to different doses of *P. gingivalis* LPS. *E. coli* *lipopolysaccharide* served as a positive control for the production of cytokines while unstimulated cells were cultured with serum free medium alone. Every cell treatment was carried out three times. After that, cell supernatants were gathered, centrifuged to get rid of any remaining cell debris and kept at -20°C. In order to generate submaximal cytokine production, circumstances identified from the early investigations were used to compare the cytokine response of gingival fibroblasts from healthy and inflamed tissues. Exposure to *E. coli* *lipopolysaccharide* served as a positive control and unstimulated cells were once more cultivated in serum-free medium. A total of 10 healthy donors who were matched by age and five donors who had been diagnosed with periodontitis underwent each cell therapy in triplicate.

## Detection of cytokines by multiplex analysis of gingival fibroblast cell culture supernatant:

Using bead-based multiplex assays, gingival fibroblast cell culture supernatants were examined for mediators. In the current investigation, a 6-plex, which includes VEGF, MCP-1, IL-6, IL-8, IL-1β, and GRO, was employed. As directed by the manufacturer, triplicate analyses of each supernatant were performed. In short, assay buffer was used to pre-wet 96-well filter plates before they were vacuum-removed. The plate was loaded with duplicate standards, controls and samples before antibody-immobilized beads were added. All background, standard and control wells received serum-free medium. After that, plates were incubated at 4°C for the entire night on a shaking platform. The next day, the liquid was vacuumed out of the wells and given two washes with wash buffer. Following the addition of detection antibodies, the wells were incubated at room temperature for one hour on a shaking platform. Following this incubation time, streptavidin-phycoerythrin was added to the wells and allowed to sit at room temperature for 30 minutes on a shaking platform. After vacuuming out all of the liquid from the wells, the wells were twice cleaned with wash buffer before the beads were re-suspended in sheath fluid for five minutes on a shaking platform. A Luminex 200TM Analyzer was then used to run each plate, gathering 50 beads per bead set and 100µL. Using xPONENT version 3.1 software (Luminex Corporation), a weighted 5-parameter logistic curve for each mediator with a range of 10,000 to 3.2 pg/ml was used to create a 6-point standard curve. Cytokine levels in human gingival fibroblast cell supernatant from both healthy and inflammatory gingival tissue after exposure to *P. gingivalis* LPS. For 24 hours, *P. gingivalis* *lipopolysaccharide* (1µg/ml) was administered to human gingival fibroblasts, while *E. coli* *lipopolysaccharide* (1.0µg/ml) served as a positive control.

## Statistical analysis:

After being exposed to various cell therapies, gingival fibroblasts generated from healthy or inflamed tissue were examined for variations in their responses using a standard two-way ANOVA and Sidak's multiple comparisons test. Statistical significance was defined as a p value of less than 0.05.

## Results:

MCP- 1 in inflamed gingiva was 56 ± 03 pg/mL, 217 ± 16 pg/mL and 4032 ± 236 pg/mL corresponding to unstimulated, *E-coli* *lipopolysaccharide* and *P. gingivalis* *lipopolysaccharide* respectively. It was greater in *P. gingivalis* *lipopolysaccharide* as compared to unstimulated, *E-coli* LPS. GRO in inflamed gingiva corresponding to Gingivalis *lipopolysaccharide* was 417± 104 pg/mL. It was greater in *P. gingivalis* *lipopolysaccharide* as compared to unstimulated, *E-coli* LPS.IL-6 in inflamed gingiva was 12 ± 02 pg/mL, 34 ± 08pg/mL and 548 ± 112 pg/mL corresponding to unstimulated, *E-coli* *lipopolysaccharide* and *P. gingivalis* *lipopolysaccharide* respectively. It was greater in *P. gingivalis* *lipopolysaccharide* as compared to unstimulated, *E-coli* LPS. IL-8 in inflamed gingiva was01± 01 pg/mL, 46± 03 pg/mL and 963± 121 pg/mL corresponding to unstimulated, *E-coli* *lipopolysaccharide* and *P. gingivalis* *lipopolysaccharide* respectively. It was greater in *P. gingivalis* *lipopolysaccharide* as compared to unstimulated, *E-coli* LPS. VEGF in inflamed gingiva was 31 ± 01 pg/mL, 34 ± 02 pg/mL and 42 ± 12 pg/mL corresponding to unstimulated, *E-coli* *lipopolysaccharide* and *P. gingivalis* *lipopolysaccharide* respectively. It was greater in *P. gingivalis* *lipopolysaccharide* as compared to unstipulated, *E-coli*- *lipopolysaccharide* (Table 1 - see PDF).

## Discussion:

While numerous studies have demonstrated that gingival fibroblasts respond to *P. gingivalis* LPS, the majority of these studies use murine cell lines, easily available gingival fibroblasts cell lines, or healthy donor cells [3, 4, 5-6]. Inflammatory gingival fibroblasts have not been widely employed in research. In studies to assess gingival fibroblasts cytokine production, the effect of *E. coli* *lipopolysaccharide* exposure is most commonly compared with the creation of inflammatory cytokines by *P. gingivalis* *lipopolysaccharide* [9, 10, 11-12]. Therefore, the present study was carried out to compare the cytokine response of primary gingival fibroblasts from healthy and inflamed gingival tissues and to evaluate whether gingival fibroblasts from inflamed gingiva are accomplished of establishing a secondary inflammatory response after exposure to *P. gingivalis* *lipopolysaccharide* *In vitro*. In our study, findings show that inflamed gingival fibroblasts react to *P. gingivalis* *lipopolysaccharide* through the production of more pleiotropic cytokines like IL-6 and many chemokines like MCP-1, IL-8 and GRO. The findings are similar to observations of other studies that showed gingival fibroblasts respond significantly to the periodontal infections [4, 5, 6, 7- 8]. Gingival fibroblasts which were once thought to be innocuous bystanders in the host defense system are now known to actively and significantly contribute to the coordination of many defense mechanisms needed to combat infection [13, 14-15]. One of *P. gingivalis*'s many virulence agents, *lipopolysaccharide* (LPS), triggers an inflammatory and metabolic response in these cells. Toll-Like Receptors (TLR) are thought to be the mechanism by which *P. gingivalis* *lipopolysaccharide* interacts with gingival fibroblasts initiating a number of intracellular signaling pathways that subsequently alter cytokine production [18, 21]. Though it lacks an intracellular signaling moiety that would allow instructions to reach the cell [16, 17-18], CD14 is likewise expressed on the cell surface by gingival fibroblasts [23, 24- 25].

In our study IL-6 in inflamed gingiva was 12 ± 02 pg/mL, 34 ± 08 pg/mL and 548 ± 112 pg/mL corresponding to unstimulated, *E-coli* *lipopolysaccharide* and *P. gingivalis* *lipopolysaccharide* respectively. It was greater in *P. gingivalis* *lipopolysaccharide* as compared to unstimulated *E-coli* LPS.IL-8 in inflamed gingiva was 01± 01 pg/mL, 46± 03 pg/mL and 963± 121 pg/mL corresponding to unstimulated, *E-coli* *lipopolysaccharide* and *P. gingivalis* *lipopolysaccharide* respectively. It was greater in *P. gingivalis* *lipopolysaccharide*as compared to unstimulated *E-coli* LPS.These observations of our study have similarity with other studies that showed inflammatory response by gingival fibroblasts against *P. gingivalis* through increased IL-6 and IL-8 production [18, 19, 20, 21, 22-23]. Cells in the gingival tissue produce a variety of pro-inflammatory mediators, including Tumor Necrosis Factor (TNF), Interleukin-1 (IL-1), IL-8 and IL-6. These cytokines contribute to the attraction and stimulation of inflammatory cells, which allows for tissue regeneration and healing in an effort to control the ensuing infection and maintain the homeostasis of the tissues [20, 21- 22]. Periodontitis develops as a result of the host's immune system reacting to the contamination of the sub gingival environment by Gram-negative microorganisms such as *Porphyromonas gingivalis* (*P. gingivalis*) and numerous other pathogenic organisms, including *Treponema denticola* and *Fusobacterium nucleatum* [21, 22-23]. *P. gingivalis* is an anaerobic bacterium that is a member of the red complex group of bacteria. It is an important microbe associated with periodontitis and related symptoms [17, 18, 19, 20, 21, 22-23]. The onset and progression of periodontitis may be influenced by the immune system's reaction to the first interaction between *P. gingivalis* and different cell types, including tissue-resident macrophages, gingival epithelial cells, gingival fibroblasts (GF), and migrating pro-inflammatory neutrophils, monocytes and macrophages [18, 19- 20]. Circulating inflammatory cells also affect the general inflammation associated with periodontitis. The risk of developing a number of chronic disorders is increased by changes in the levels of inflammatory mediators in the bloodstream brought on by sub gingival contamination of periodontal tissue by pathogenic bacteria [26- 27].

## Conclusion:

There was a significant amount of cytokine release from gingival fibroblasts exposed to periodontal pathogens.
